# Normalization Challenges Across Adipocyte Differentiation and Lipid-Modulating Treatments: Identifying Reliable Housekeeping Genes

**DOI:** 10.3390/ijms27031369

**Published:** 2026-01-29

**Authors:** Zhenya Ivanova, Valeria Petrova, Toncho Penev, Natalia Grigorova

**Affiliations:** 1 Department of Pharmacology, Animal Physiology, Biochemistry and Chemistry, Faculty of Veterinary Medicine, Trakia University, 6000 Stara Zagora, Bulgaria; zhenya.ivanova.12@trakia-uni.bg (Z.I.); valeriya.petrova@trakia-uni.bg (V.P.); 2 Department of Ecology and Animal Hygiene, Faculty of Agriculture, Trakia University, 6000 Stara Zagora, Bulgaria; toncho.penev@trakia-uni.bg

**Keywords:** adipogenesis, RT-qPCR gene expression, reference gene stability, 3T3-L1, algorithm-based normalization

## Abstract

Accurate normalization of RT-qPCR data requires selecting stable internal control genes, particularly in models characterized by dynamic metabolic transitions, such as 3T3-L1 adipocytes. The current study compares the expression stability of nine widely used housekeeping genes (HKGs) (peptidylprolyl isomerase A (*Ppia*), glyceraldehyde-3-phosphate dehydrogenase (*Gapdh*), beta-2 microglobulin (*B2M*), ribosomal protein, large, P0 (*36b4*), hydroxymethylbilane synthase (*Hmbs*), hypoxanthine guanine phosphoribosyl transferase (*Hprt*), tyrosine 3-monooxygenase/tryptophan 5-monooxygenase activation protein, zeta polypeptide (*Ywhaz*), 18S ribosomal RNA (*18S*), and β-actin (*Actb*)) across key stages of differentiation (days 0, 9, and 18) and under treatments with palmitic acid and docosahexaenoic acid. Stability was assessed using four classical algorithms—geNorm, NormFinder, BestKeeper, and RefFinder—supplemented by the ΔCt method, conventional statistical testing, correlation, and regression analysis relative to two target genes, fatty acid-binding protein 4 (*Fabp4*) and sterol regulatory element binding transcription factor 1 (*Srebf1*). The obtained data indicate that no single HKG remains universally stable across these experimental conditions, and the expression of traditionally used reference genes (*Gapdh*, *Actb*, *Hprt*, *18S*) is highly influenced by both the stage of adipogenesis and exposure to lipid-modulating factors. In contrast, *Ppia*, *36b4*, and *B2M*—despite some of them being underestimated in use as references—consistently display the lowest variability across most analytical tools, forming a reliable and functionally diverse normalization panel. It should be noted that our initial stability assessment revealed apparent discrepancies among mathematical evaluation methods, emphasizing the need for a holistic, multiple-level approach strategy. The applied combination of algorithmic and statistical methods provides a more rigorous and objective framework for assessing the stability of reference genes, which is highly recommended in such a complex adipocyte-based model.

## 1. Introduction

Obesity and obesity-provoked metabolic disorders, such as dyslipidemia, insulin resistance, type 2 diabetes, and hepatic steatosis, represent some of the most critical and extensively researched health challenges [1,2,3,4]. Over a third of the world’s population is overweight or obese, and unfortunately, there is a clear, alarming trend of expansion across all age groups. In general, these conditions represent a complex interaction between genetic, epigenetic, hormonal, and environmental factors that disrupt the body’s energy balance. However, an increasingly stressful and sedentary lifestyle triggers similar conditions even in individuals who are not prone to them [5,6]. Although this topic has been explored for decades, recent years have seen a steady rise in studies focused on clarifying the detailed mechanisms of adipogenesis [7,8] and examining how various drugs, nutritional supplements, and bioactive molecules influence the metabolic regulation of adipocytes [9,10,11]. The 3T3-L1 cell line has become a classic model for studying cell differentiation and lipid accumulation. It is widely used in functional research related to obesity, insulin resistance, and lipid homeostasis [12,13,14,15,16]. This model offers the opportunity to precisely track the morphological, biochemical, and molecular changes associated with adipogenesis, as well as to assess the effects of various pharmacological agents, nutrients, and biologically active molecules on the metabolic activity of adipocytes [17,18,19]. Its reproducible experimental conditions have made the 3T3-L1 cell line a key tool in modern in vitro investigations of fat biology and metabolic homeostasis [12,20]. However, the dynamic changes occurring during differentiation introduce specific methodological challenges for molecular analyses, particularly for techniques such as reverse transcription quantitative polymerase chain reaction (RT-qPCR), where data interpretation is highly sensitive to experimental variability [9,14].

RT-qPCR, one of the most widely used molecular methods for gene expression analysis, enables precise quantification of gene expression but requires highly reliable normalization to ensure the accuracy and comparability of results. In this respect, selecting suitable housekeeping genes (HKGs) that remain consistently expressed across various experimental conditions is a crucial step for obtaining reliable results [14,21,22]. During adipogenesis, cells undergo dramatic shifts in metabolic activity, morphology, cytoskeletal organization, and overall gene expression. As a result, there is considerable variability in the expression of traditionally used HKGs, such as glyceraldehyde-3-phosphate dehydrogenase (*Gapdh*) and β-actin (*Actb*), as their levels are highly dependent on lipogenic level, cytoskeletal remodeling, and overall energy metabolism [22,23,24,25]. If we add strong antiadipogenic and lipid-modulating agents to this already complex scenario, the stability of many conventional reference genes may be further compromised. Moreover, in precise data analysis, it is recommended to avoid using highly expressed genes, such as 18S ribosomal RNA (*18S*), if their Ct value differs by more than 5 cycles from that of the target gene. This discrepancy can complicate baseline correction and generate errors related to amplification efficiency across a wide range of Ct differences [26,27,28]. As a result, a critical question arises about which housekeeping genes remain reliable internal controls in experiments involving both undifferentiated and mature adipocytes, as well as various lipid-modulating treatments. Moreover, what is the minimal panel of HKGs in this case? These questions form the basis of the present study’s aim to evaluate the stability of nine commonly used reference genes: peptidylprolyl isomerase A (*Ppia*), *Gapdh*, beta-2 microglobulin (*B2M*), ribosomal protein, large, P0 (*36b4*), hydroxymethylbilane synthase (*Hmbs*), hypoxanthine guanine phosphoribosyl transferase (*Hprt*), tyrosine 3-monooxygenase/tryptophan 5-monooxygenase activation protein, zeta polypeptide (*Ywhaz*), *18S* and *Actb*, which differ in their biological functions, cellular localizations, and sensitivities to metabolic changes. The primary focus was on identifying a robust panel of internal control genes to ensure accurate and reproducible normalization of RT-qPCR data in the 3T3-L1 adipogenesis model.

The analysis encompasses various stages of overall differentiation, ranging from preadipocytes to mature adipocytes, along with lipomodulating agent treatments such as palmitic acid (PA) or docosahexaenoic acid (DHA), which have diverse biological effects. Besides the classical tools such as geNorm [29], NormFinder [30], BestKeeper [28], and RefFinder [31], the gene stability was also evaluated via the ΔCt method, conventional statistical testing, correlation, and regression analysis relative to fatty acid-binding protein 4 (*Fabp4*) and sterol regulatory element binding transcription factor 1 (*Srebf1*), as specific target genes. The applied strategy, which integrates algorithmic and statistical methods of analysis, aims to provide a robust approach for identifying a stable reference gene panel for reliable RT-qPCR analysis in this complex adipocyte model.

## 2. Results

### 2.1. Intracellular Neutral Lipid Accumulation Among the Studied Groups

**Figure 1:**
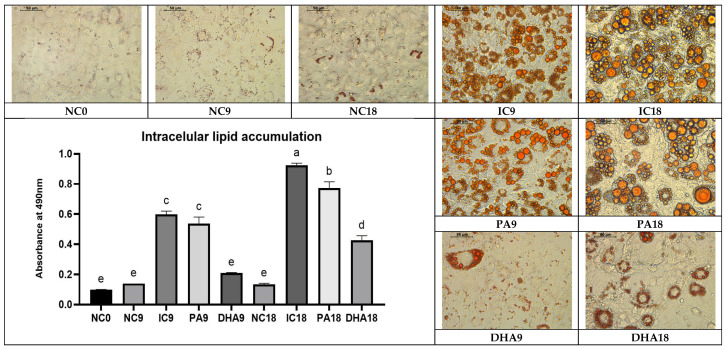
Oil Red O (ORO) staining and quantification of intracellular lipid accumulation in 3T3-L1 cells under differentiation and lipid-modulating treatments. Representative ORO-stained images of non-induced controls (NC0, NC9, NC18), induced controls (IC9, IC18), and cells treated with 60 µM palmitic acid (PA9, PA18) or 60 µM docosahexaenoic acid (DHA9, DHA18). Quantification of lipid accumulation based on absorbance of isopropanol-extracted ORO at 490 nm. Images were acquired using a Leica DMi1 Inverted Microscope (Wetzlar, Germany) equipped with a 5-megapixel-resolution camera and the Leica Application Suite Core software (V4.12) (Heerbrugg, Switzerland) (40× magnification, bar = 50 µm). Statistical significance was assessed using Tukey’s test; groups not sharing a letter differ at *p* < 0.05.

Oil Red O staining images and subsequent quantitative analysis of intracellular lipid accumulation, presented in Figure 1, confirmed the successful adipogenic differentiation of 3T3-L1 cells. As shown, undifferentiated control preadipocytes (NC0) exhibited minimal lipid accumulation, a characteristic of preadipocytes. After 9 days of adipogenic induction, a significant increase in absorbance at 490 nm was observed in the differentiated adipocytes (positive control) (IC9). This result also corresponded to the microscopic images, in which the cells acquired the typical morphology of early adipocytes, including the appearance of numerous lipid droplets. On day 18, the differentiation in IC18 was even more pronounced: quantitative data demonstrated the highest levels of lipid accumulation, along with microscopic images showing large, confluent lipid vacuoles, a classic feature of mature adipocytes. Supplementation of PA during adipogenesis (PA9 and PA18) maintained lipid accumulation at almost the same levels as fully induced, mature adipocytes in IC groups, while treatment with DHA resulted in its marked suppression. The absorbance at 490 nm in the DHA group was significantly lower compared to controls on the corresponding days, and microscopic images clearly showed a reduced number of differentiated cells.

### 2.2. Classical Reference Genes Analyses

#### 2.2.1. BestKeeper Algorithm

The analysis of descriptive statistics in the BestKeeper tool version 1 (Table 1) revealed distinct differences in stability among the candidate reference genes. *Hprt* demonstrated the lowest variability, with a coefficient of variation (CV) = 1.63% and a standard deviation (SD) = 0.47, which is below the generally accepted threshold for high stability (CV < 2%). *Ywhaz*, *Ppia*, *Gapdh*, *36b4*, and *B2M* also showed relatively low variability (CV between 3% and 5%), suggesting moderate to good stability. For them, the range between the minimum and maximum Ct values was limited, which further supports their acceptable expression stability. *Actb* had CVs slightly above 4–5%, which could also place it in the category of moderately stable genes. Their variability was not critically elevated, but still higher compared to the candidates mentioned above. *Hmbs* and *18S* were outlined as the most unstable genes, with the highest SD (1.38 and 1.46) and CV (9.48% and 10.03%, respectively). The difference between their minimum and maximum Ct values was more than 5 Ct, clearly indicating that *Hmbs* gene expression varied greatly between conditions.

Correlation analysis against the BestKeeper index (BK), presented in Table 2, revealed that *Hmbs*, *Ppia*, *18S*, *36b4*, and *B2M* exhibited a nearly linear association with the BestKeeper index (r > 0.94), indicating high consistency in their expression compared to the overall Ct profile of the panel. Slope analysis further supported the stability of *Actb*, *Ppia*, *36b4*, and *B2M* (slope ≈ 1), but raised questions about the stability of *Hmbs* and *18S* (slope = 1.424 and 1.521, respectively). *Ywhaz*, *Gapdh*, and *Hprt* showed a moderate to weak correlation with the BK. The target genes *Srebf1* and *Fabp4* correlated negatively (r = −0.472 and r = −0.538), as expected, since their expression changes dramatically during adipogenesis.

#### 2.2.2. Stability Values Evaluated via Normfinder and geNorm Excel-Based Tools

**Figure 2:**
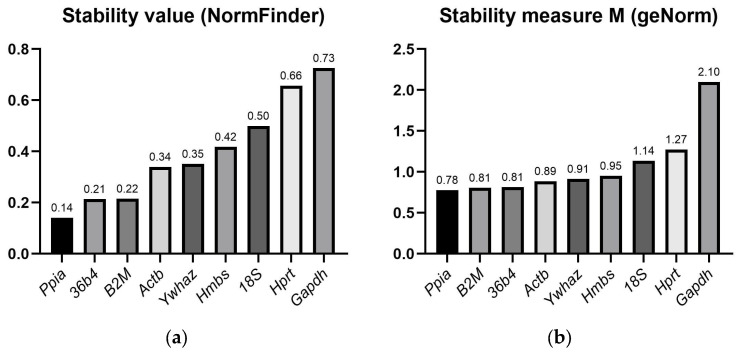
Comparative stability values and ranking of reference genes determined by (**a**) NormFinder and (**b**) geNorm.

The results presented in Figure 2 display the stability ranking of the tested HKGs, as determined by the most widely used tools: NormFinder and geNorm. NormFinder identified *Ppia* as a gene with the best stability value and also recommended *Ppia* and *36b4* as a stable combination. Based on the average M-values calculated with geNorm v3.5 (Figure 2b), all candidate reference genes—with the exception of *Gapdh* (M = 2.10)—met the threshold criteria for acceptable stability by 1.5. Among them, *Ppia* demonstrated the highest expression stability, ranking it as the most suitable HKG for normalization, closely followed by *B2M*, *36b4*, *Actb*, and *Ywhaz*.

#### 2.2.3. Summarized RefFinder Analyses

**Figure 3:**
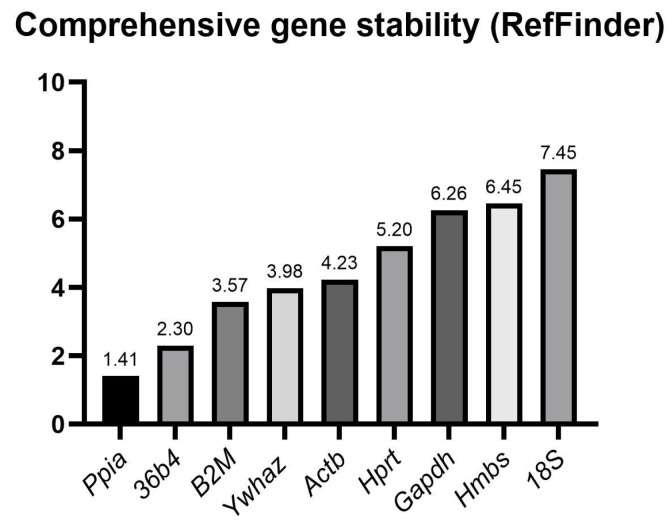
Summarized ranking evaluation of investigated reference genes assessed by the RefFinder tool. Data were obtained from nine experimental groups, each represented by five biological replicates (n = 45 per gene). Lower values indicate higher expression stability.

The combined approach in the RefFinder analyses (Figure 3), which evaluates the mean rank among geNorm, NormFinder, BestKeeper, and ΔCt methods, distinguished four levels of stability using the tested HKGs. The highest stability was demonstrated in *Ppia* and *36b4*, followed by *B2M* and *Ywhaz*; *Actb* and *Hprt* were also stable. In contrast, *Gapdh*, *Hmbs,* and *18S* were the most unstable genes.

### 2.3. Statistical Analyses (GraphPad)

#### 2.3.1. Ct Value Distribution and Basal Expression Levels of Candidate Reference Genes

**Figure 4:**
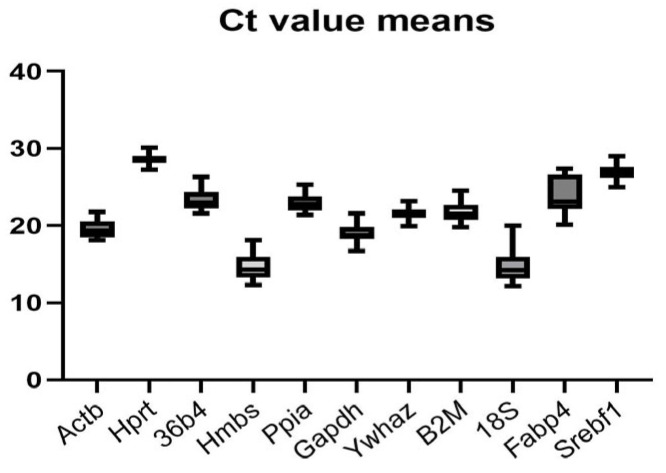
Baseline expression levels of candidate reference genes are illustrated through the distribution of cycle threshold (Ct) values across all experimental conditions. The horizontal line within each box represents the median Ct value, while the box borders indicate the first and third quartiles. The bars represent the full range of observed values.

The distribution of Ct values demonstrates apparent differences in basal expression levels among the tested genes (Figure 4). *18S* and *Hmbs* show the lowest Ct values, while *Hprt* shows the highest. *18S* and *Hmbs* differ from the mean Ct value of the target genes (*Fabp4* and *Srebf1*) by more than ten cycles, indicating their inadequacy as a reference gene. The rest of the analyzed control genes cluster within a mid-range Ct interval, suggesting moderate and relatively comparable expression levels. As expected, the adipogenic marker *Fabp4* displays the widest Ct distribution, consistent with its strong upregulation during differentiation.

#### 2.3.2. Z-Score Expression Profiling of Candidate HKGs

**Figure 5:**
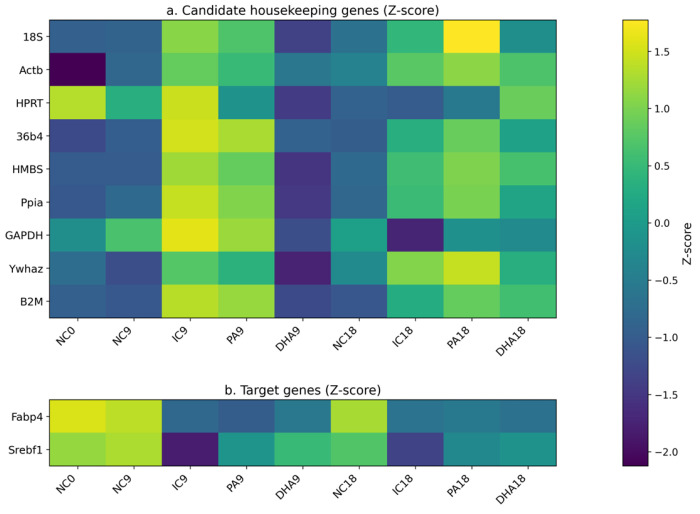
Heatmap image of Z-score-based expression: (**a**) of each candidate HKGs and (**b**) of a target (*Fabp4* and *Srebf1*) gene across differentiation and lipid-modulating conditions in 3T3-L1 cells. Warmer colors indicate higher relative expression (positive Z-scores), whereas cooler colors reflect lower relative expression (negative Z-scores). Z-score for each gene was assessed in Excel by applying the following formula: Z = (Ct − Mean Ct)/SD. Heatmaps were generated using Python version 3.10 (matplotlib) with assistance from an AI tool (ChatGPT 5.2, OpenAI) for code drafting. Final visualization parameters were selected and approved by the authors.

The heatmap displays the standardized (Z-score) mean expression values of the studied HKGs and the target adipogenic markers *Fabp4* and *Srebf1* across the different experimental conditions (Figure 5). This visualization highlights condition-dependent fluctuations in the stability of all reference genes. The gene expression of most of these genes (*18S*, *Actb*, *36b4*, *Hmbs*, *Ppia*, *Gapdh*, *Ywhaz*, and *B2M*) is positively affected in mature adipocytes (IC9, PA9, IC18, and PA18). Only in *Hprt*, no direct relationship between its expression and adipogenic progression was established.

#### 2.3.3. Statistical Comparison of Raw Ct Values Across Experimental Groups

**Figure 6:**
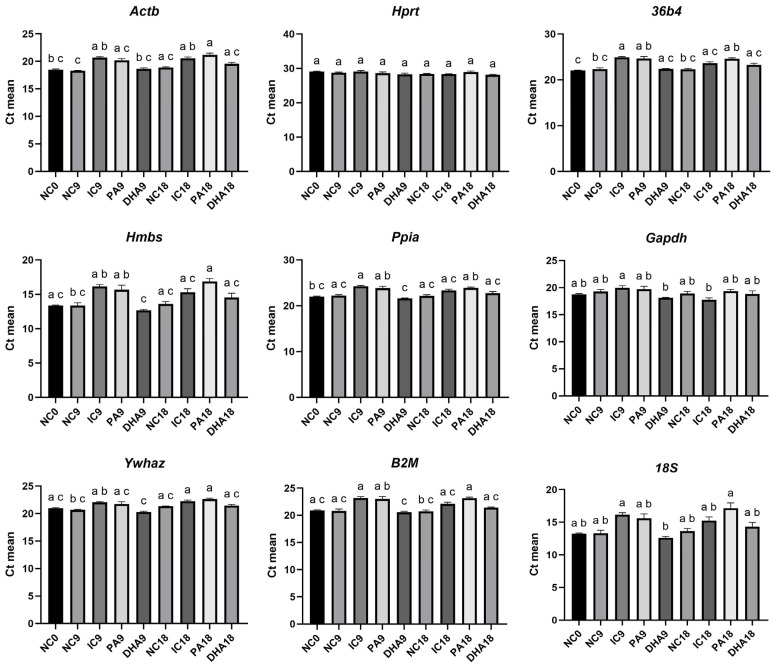
Intergroup comparison of raw Ct values (mean ± SEM) for each candidate reference gene in 3T3-L1 cells. Significant differences between groups, identified using the Kruskal–Wallis test, are denoted by distinct letters above the bars (*p* < 0.05).

To quantitatively present the observed changes depicted in the heatmap image, we conducted a classical non-parametric Kruskal–Wallis test and determined the statistical differences in the raw Ct values among the individual experimental groups. The results, presented in Figure 6 align completely with the graphical visualization: the expression levels of all candidate reference genes were significantly affected by both adipogenic induction and the applied lipid-modulating factors. Only for the *Hprt* gene were no statistically significant differences found.

#### 2.3.4. Pairwise ΔCt Stability Evaluation of Candidate HKGs

As an additional validation step, the stability of the candidate reference genes was examined using the ΔCt comparison method of Silver et al. [32], which evaluates each gene by determining the extent of variation in its pairwise ΔCt comparisons with all other candidates (Table 3). Our calculations identified *Ppia*, *36b4*, *B2M*, *Actb*, and *Ywhaz* as the most stable genes, with mean SD values ranging from 0.62 to 0.71. In contrast, *Hprt*, *Gapdh*, and *Hmbs* showed the highest variability, with SD values close to or exceeding 1, confirming their lower stability.

#### 2.3.5. Correlation Analyses Between Each HKG with *Fabp4* or *Srebf1*

Statistically, correlations of r ≥ ±0.7 are considered strong, moderate correlations typically fall between r = ±0.5 and ±0.7, weak fall between r = ±0.3 and ±0.5, and correlations below ±0.3 are regarded as negligible. The results presented in Table 4 and Table 5 show that none of the analyzed HKGs exhibit a strong correlation with *Fabp4 or Srebf1*. *36b4*, *Actb*, *B2M*, and *18S* display moderate correlations, which may limit their suitability as validation controls. The correlations observed for *Hmbs*, *Ppia*, and *Ywhaz* are weaker, whereas *Hprt* and *Gapdh* show no statistically significant correlation with the *Fabp4* and *Srebf1* Ct pattern.

#### 2.3.6. Integrated Stability Ranking of Candidate Reference Genes

The data in Table 6 summarizes the results obtained in the current study. Based on the analyzed variability, simplified stability rankings were formed. As a result of the complex experimental model, a fully uniform ranking across all methods could not be generated. However, several genes consistently appear among the top three most stable candidates: *Ppia* (in all 6 analyses), *36b4* (in 4 out of 6 analyses), and *B2M* (in 4 out of 6 analyses). These genes demonstrate sufficient reliability to be used in combination as a two- or three-gene normalization panel.

#### 2.3.7. Normalization of Fabp4 ΔCt Values Across All Tested Reference Genes

**Figure 7:**
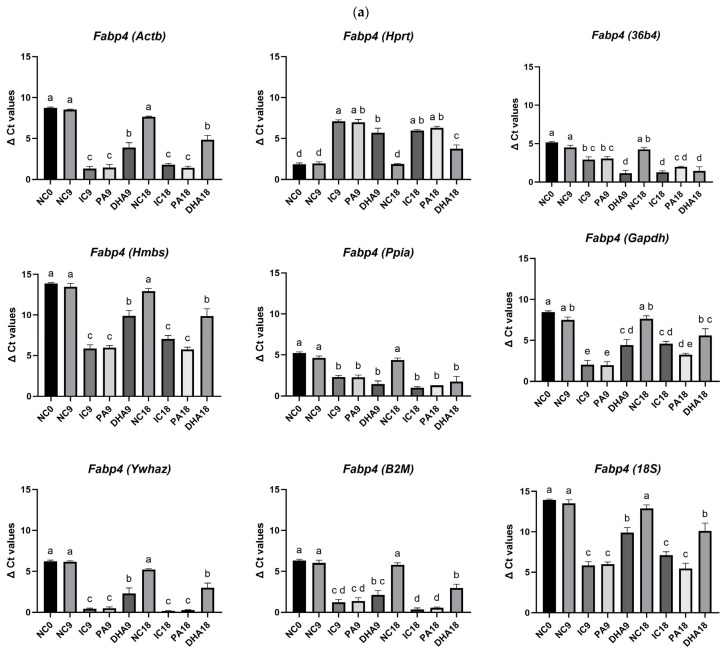

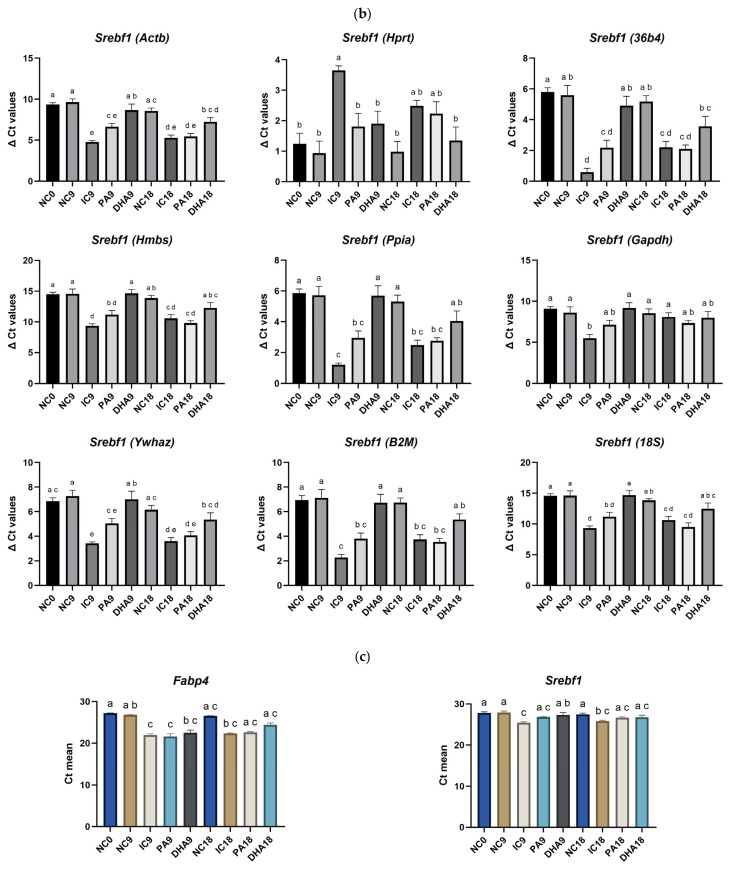
ΔCt values of (**a**) *Fabp4* and (**b**) *Srebf1* genes normalized to each candidate reference gene (*Actb*, *Hprt*, *36b4*, *Hmbs*, *Ppia*, *Gapdh*, *Ywhaz*, *B2M*, *18S*), shown in comparison with their raw Ct expression patterns (**c**). Data are presented as mean ± SEM. Different letters above the bars indicate statistically significant differences at *p* < 0.05 using Tukey’s test.

To further support our statement that *Ppia*, *36b4*, and *B2M* are consistent enough to be included in the normalization panel, we performed reference adjustment of *Fabp4* (Figure 7a) *and Srebf1* (Figure 7b) against each of the tested control genes. The analysis presented in Figure 7 revealed apparent differences in the assessment of expression dynamics depending on the internal control used. In eight of the tested controls (*Actb*, *36b4*, *Hmbs*, *Ppia*, *Gapdh*, *Ywhaz*, *B2M*, *18S*), the overall pattern of normalized *Fabp4* and *Srebf1* gene expression closely resembled that observed in the row Ct data: undifferentiated cells (NC0, NC9, NC18) exhibited the highest ΔCt values, corresponding to low *Fabp4* and *Srebf1* expression, whereas differentiated and lipid-modulated groups, especially PA-treated cells, demonstrated significantly lower ΔCt values, indicative of increased adipogenic activity.

However, the amplitude and statistical evaluation of these changes varied significantly between the individual reference genes. For the genes indicated as the most stable in Table 6, consistent and biologically logical differences between the groups were observed, especially in *Fabp4* gene expression, normalized to *Ppia* and *36b4*. In contrast, the use of *Hprt* would lead to substantial changes in the statistical differences and inconsistencies in interpreting the effects.

## 3. Discussion

Accurate normalization is essential for reliable RT-qPCR analysis, but the selection of appropriate HKGs in adipogenic models remains a significant methodological challenge. According to the available literature to date, it is evident that there is no unified model, particularly in highly dynamic cells such as differentiating adipocytes [22,33,34,35]. The present study systematically evaluated nine commonly used reference genes in a classical 3T3-L1 model across three stages of differentiation (days 0, 9, and 18), as well as under exposure to lipid-modulating compounds (DHA and PA). These conditions induce significant alterations in cellular metabolism and overall phenotype, which inevitably affect the expression of most of the commonly used HKGs. Therefore, the use of a single reference gene is insufficient and carries a considerable risk of incorrect normalization [22]. To demonstrate this, in the present study, we normalized the selected target genes, *Fabp4* and *Srebf1*, against each of the tested HKGs, and the resulting discrepancies were divergent. Moreover, the heatmap analysis together with the statistical evaluation of intergroup variability clearly demonstrates instability across the examined conditions, with none of the HKGs maintaining consistent expression under all experimental conditions. Therefore, in this type of investigation, normalization to a single reference gene—regardless of how widely it may be recommended in the literature—is impractical and would result in large errors in data interpretation. Current minimal standards for generating accurate RT-qPCR results require not only precise experimental execution and analytical rigor, but also normalization of target genes to a reliable and stable set of at least three HKGs. This approach ensures the robustness of RT-qPCR analysis even in highly dynamic biological models such as adipogenesis [27,36,37]. It is further advisable that the selected internal controls represent different biological functions to minimize fluctuations in expression. Additionally, normalization based on the geometric mean rather than the arithmetic mean of the HKG panel is recommended, as this prevents any single reference gene from disproportionately influencing the final normalization factor [29].

In our study, we evaluated the stability of nine commonly used HKGs that differ in both their biological functions and their sensitivity to the metabolic shifts characteristic of adipogenesis. Among these, *Gapdh*, a key glycolytic enzyme, is frequently used as a single reference gene. Still, recent studies report substantial variability in its expression due to the enhanced glycolytic activity of mature adipocytes and lipid-induced metabolic changes, raising concerns about its reliability as an internal control. Similarly, *Actb*, widely used as a single control, encodes β-actin, a structural protein that is strongly influenced by cytoskeletal remodeling during adipogenesis. B2M, encoding β2-microglobulin, is a small non-glycosylated protein (~12 kDa) and an essential structural component of major histocompatibility complex class I (MHC I) molecules [38]. Because MHC I is ubiquitously expressed in nearly all nucleated cells, *B2M* has traditionally been used as a reference gene in RT-qPCR analyses [39]. However, an increasing body of evidence indicates that its expression is not constant across all contexts: *B2M* is sensitive to inflammatory cytokines, hypoxia, lipotoxicity, and other forms of metabolic stress. In adipocyte models, including 3T3-L1 cells, these stimuli modulate the antigen-presenting machinery, leading to elevated *B2M* expression during differentiation or inflammatory activation [33]. According to the literature, Hmbs, an enzyme involved in heme biosynthesis, and Hprt, a key component of the purine salvage pathway, generally display moderate but acceptable expression stability. Although Hmbs may be influenced by oxidative stress and Hprt by changes in cellular proliferation, their variability under standard adipogenic conditions remains relatively limited. This makes them suitable as supplementary reference genes in 3T3-L1 adipocyte models. 18S rRNA is a structural component of the 40S ribosomal subunit and plays a key role in mRNA translation. Although it is commonly used as a housekeeping gene in adipocyte studies due to its apparent expression stability [22], its suitability remains debatable because its very high abundance relative to mRNA targets can introduce normalization bias [37]. Interestingly, genes such as *36b4*, which encodes the ribosomal protein P0 of the 60S subunit; *Ppia*, encoding cyclophilin A involved in protein folding [40]; and *Ywhaz*, which encodes the protein 14-3-3ζ—a regulatory gene participating in insulin signaling and multiple cellular pathways [41,42], typically show exceptionally stable expression in adipocytes but are used far less frequently as normalization controls. Their roles in fundamental and constitutive cellular processes—such as translation, proteostasis, and signaling integration—likely contribute to their maintained transcript stability, even during pronounced shifts in cellular phenotype. Consequently, these genes exhibit minimal variability throughout adipogenesis and under metabolic stress [43,44].

Based solely on their biological roles, it is evident that the experimental conditions in our model inevitably introduce some degree of variation in the expression of nearly all conventionally used internal controls. This underscores the importance of careful selection and empirical validation when establishing an appropriate panel of reference genes.

Even when candidate reference genes are carefully preselected, and RT-qPCR assays are performed accurately, with optimal reaction efficiency, researchers often face a significant challenge: different software and mathematical algorithms frequently produce divergent assessments of gene stability. The application of classical statistical approaches can further complicate interpretation, as these methods also do not always yield a unified ranking. As a result, the selection of an appropriate panel of HKGs is sometimes performed partially “blindly”, especially when no clear consensus emerges across algorithms.

At first glance, however, the bioinformatic tools and classical ones in our evaluations produced apparently divergent outcomes. The three most commonly used algorithmic methods—NormFinder, geNorm, and RefFinder—calculated similar rankings, consistently identifying *Ppia*, *36b4*, *B2M*, *Ywhaz*, and *Actb* as the most stable genes, while reporting substantial instability for *Gapdh*, *Hprt*, *18S*, and *Hmbs*. In contrast, descriptive analyses performed with BestKeeper, which rank genes based on variability measures (SD and CV), highlighted *Hprt*, *Ywhaz*, *Ppia*, and *Gapdh* as the most stable candidates, while excluding *Hmbs* and *36b4* from the stable group.

Such discrepancies are expected and arise from both the complexity of the experimental model and the fundamentally different mathematical principles underlying these two categories of methods. Algorithm-based tools prioritize internal stability within the reference gene set, whereas classical statistical analyses emphasize absolute differences between experimental groups, independent of how other housekeeping genes behave. Consequently, a gene may appear statistically stable (e.g., *Hprt*), as it is the only gene without significant intergroup differences, yet still exhibits high relative variability when evaluated within multi-gene normalization algorithms. This illustrates the limitations of relying solely on either statistical tests or algorithmic rankings alone.

As an independent strategy for assessing the expression stability of the reference genes, we applied the method of Silver et al. [32], which is based on pairwise ΔCt comparisons between reference genes. Implementing this approach in our model allowed us to evaluate not individual genes, but the mutual consistency among every possible gene pair. In this way, the ΔCt analysis provides an independent, experimentally grounded assessment that does not rely on algorithmic assumptions but instead reflects the actual behavior of the genes. The results obtained closely correspond to those derived from NormFinder, geNorm, and RefFinder. The least stable genes identified were *Hprt*, *Gapdh*, *18S*, and *Hmbs*, whereas the most stable were *Ppia*, *36b4*, and *B2M*.

To ensure a more robust and well-supported selection of internal controls, we additionally performed regression analysis in BestKeeper and correlation analysis in GraphPad, incorporating *Fabp4* and *Srebf1* as the target genes in both models. The choice of an appropriate target gene is a critical component of reference gene validation, as it must exhibit clear context-dependent variability that reflects the true biological changes occurring within the system. *Fabp4* represents an optimal target gene in our model. It is a well-established adipogenic marker in 3T3-L1 cells, with expression that increases markedly during the transition from preadipocytes to mature adipocytes and responds sensitively to lipid modulators such as palmitate and DHA. As an intracellular fatty acid transporter, *Fabp4* directly reflects dynamic shifts in lipid metabolism and therefore displays the expected high variability across the experimental conditions. *Srebf1* was included as a second target gene to introduce complementary biological variability. As a central regulator of lipogenesis and adipocyte maturation, *Srebf1* captures regulatory changes distinct from the lipid transport-related function of *Fabp4*, enabling a broader and more balanced evaluation of reference gene performance.

Stable reference genes should remain unaffected by the biological factors influencing the target gene. For this reason, correlation analysis against the target gene should play a key role in defining the normalization panel, although this approach is seldom applied in practice. In our study, none of the analyzed HKGs exhibited a strong correlation with *Fabp4* or *Srebf1*, suggesting that they do not artificially mask the true variability of this dynamically regulated target gene in the RT-qPCR analysis. Nevertheless, for genes showing weak to moderate correlations—*Hmbs*, *Ppia*, *Ywhaz*, *36b4*, *Actb*, and *B2M*—additional evaluation and validation were required before they can be confidently incorporated as internal controls.

The combined regression analysis in BestKeeper provides an even more precise and reliable basis for refining the selection of our reference gene panel. It is well established that the overall HKG panel (BestKeeper index) should not correlate with the target gene, and that for an individual HKG to be included in the internal control set, it must exhibit a correlation coefficient of at least 0.9 with the BK index. In our dataset, all evaluated genes met these criteria except *Hprt* and *Gapdh*.

Integrating all applied approaches—both algorithmic and experimentally based—enabled the generation of a reliable, ranked assessment of the stability of individual housekeeping genes. In the final panel, we included those genes that consistently ranked among the most stable across the majority of evaluation methods. Based on this, we recommend a panel composed of three reference genes (*Ppia*, *B2M*, and *36b4*), which collectively represent diverse biological functions and thereby minimize the risk of co-regulation. This combination provides more accurate and reliable normalization for RT-qPCR analysis of 3T3-L1 adipocytes under conditions of differentiation and lipid interventions.

Nevertheless, our analysis has certain limitations. Reference gene stability is context-dependent, and the results apply specifically to the adipogenic model, time points, and lipid modulators used in this study. Other metabolic or inflammatory stimuli were not included, nor were additional cell lines or primary adipocytes, including human models, which limits the generalizability of the findings beyond the present experimental design. Additionally, differences in RT-qPCR reagents and amplification conditions may further contribute to variability in reference gene stability across studies.

## 4. Materials and Methods

### 4.1. Experimental Design and Cell Treatments

#### 4.1.1. Cell Line and Culture Conditions

3T3-L1 mouse preadipocytes (CRL-3242, ATCC, Washington, DC, USA) were thawed and expanded in T75 culture flasks following standard maintenance procedures. Cells were grown in high-glucose 4.5 g/L Dulbecco’s modified Eagle’s medium (DMEM-HG) supplemented with 10% fetal bovine serum (FBS) and 1% antibiotic mixture (penicillin G, streptomycin, amphotericin B)—control medium (CM)—at 37 °C in a humidified 5% CO_2_ incubator. After two passages, cultures were seeded into 12-well plates (Corning, Costar) at an initial density of 1 × 10^4^ cells/mL and allowed to proliferate until full confluence was achieved. Once the monolayers reached 100% confluence, cells were maintained in CM for 24 h to synchronize the cell cycle prior to the induction of differentiation (Day 0—growth arrest). 3T3-L1 cells were grown in CM throughout the experiment and served as negative control groups (NC0, NC9, and NC18).

#### 4.1.2. Adipogenic Differentiation and Fatty Acid Treatments (Days 1–18)

Adipogenic differentiation was initiated on Day 1 by replacing CM with adipogenic induction medium (IM) for 48 h. IM consisted of DMEM supplemented with 10 μg/mL insulin, 0.1 mM 3-isobutyl-1-methylxanthine (IBMX), 1 μM dexamethasone, and 0.05 mM indomethacin, all purchased from Sigma-Aldrich Chemie GmbH (Merck KGaA, Darmstadt, Germany). According to the ATCC protocol, the 3T3-L1 cell line reaches full maturation on Day 8. Therefore, after 48 h of chemical induction on Day 2, the cells were cultured in maintenance medium (MM), composed of CM supplemented with 10 μg/mL insulin, for an additional 7 (IC9) or 16 days (IC18). The lipid-modulated groups-PA and DHA-were exposed to 60 μM of the corresponding fatty acid (PA, P5585, Sigma-Aldrich Chemie GmbH or DHA, D2534, Sigma-Aldrich Chemie GmbH) from Day 1 to Day 9 of the adipogenesis (PA9, DHA9). Then they were kept in MM for further maturation (PA18, DHA18). The dissolving stepwise procedure is detailed by [45].

The control and experimental groups included in the current study are summarized in Table 7.

For each experimental group, cells cultured in 12-well plates were used both for Oil Red O staining to assess lipid accumulation and for mRNA extraction for subsequent RT-qPCR analysis.

### 4.2. Quantification of Intracellular Lipid Deposition

Neutral lipid accumulation in mature adipocytes from all experimental groups was evaluated through Oil Red O staining, followed by dye extraction and spectrophotometric quantification, as described by Yang et al. [46]. Briefly, after removal of the culture supernatants, the cells were washed with PBS, fixed in 10% neutral buffered formalin, rinsed with 60% isopropanol, and subsequently stained with a freshly prepared Oil Red O (Sigma-Aldrich, St. Louis, MO, USA) working solution. The stained adipocytes were observed under a Leica inverted microscope equipped with a 5-megapixel DMi1 camera to document the formation of intracellular lipid droplets. To quantify the accumulated lipids, the bound dye was extracted with 100% isopropanol, and the absorbance of the resulting solution was measured at 490 nm using a Synergy™ LX Multi-Mode Microplate Reader (BioTek Instruments, Inc., Winooski, VT, USA). The obtained values were presented relative to IC after the spontaneous adipogenesis measured in NC was excluded.

### 4.3. Gene Expression Analysis

#### 4.3.1. mRNA Isolation and Conversion to Stable cDNA for Storage

mRNA was isolated with the RNeasy Mini Lipid Tissue Kit (Qiagen GmbH, Hilden, Germany) according to the manufacturer’s instructions. The concentration and purity of the extracted RNA were determined spectrophotometrically by measuring absorbance at 260 and 280 nm using a Synergy™ LX Multi-Mode Microplate Reader with a Take3 Microvolume Plate (BioTek Instruments, Inc., Winooski, VT, USA). After adjusting all samples to an equal RNA concentration, reverse transcription was carried out using the RevertAid First Strand cDNA Synthesis Kit (Thermo Scientific, Waltham, MA, USA), and the generated cDNA samples were subsequently stored at −20 °C.

#### 4.3.2. Primer Design

Primer pairs for the housekeeping and target genes, with the exception of 18S, were designed and validated using a combination of web-available bioinformatic tools. Most primers were generated through NCBI resources and Primer3, following the design criteria outlined in the SYBR Green qPCR Master Mix guidelines. Primer specificity was confirmed using Primer-BLAST (NCBI) https://www.ncbi.nlm.nih.gov/tools/primer-blast (accessed on 21 October 2024), Primer3plus (version: 3.2.5) (https://www.bioinformatics.nl/cgi-bin/primer3plus/primer3plus.cgi, accessed on 21 October 2024), and product characteristics—including expected amplicon length, melting temperatures, and predicted melting curves—were evaluated with Primer3 (version 4.1.0) (https://primer3.ut.ee/, accessed on 21 October 2024) and uMelt Quartz (version 3.6.2) (https://www.dna-utah.org/umelt/quartz/um.php, accessed on 22 October 2024). A temperature-gradient assay was subsequently performed to determine the optimal annealing temperature, which consistently fell within the range of 58–61 °C, close to the standard recommendation of 60 °C. The final primer sequences used in the study are presented in Table 8. The *18S* primer sequence was originally described by Arnhold et al. [47].

#### 4.3.3. RT-qPCR Assay

RT-qPCR was carried out using a SYBR Green–based two-step protocol with the KAPA SYBR^®^ Fast qPCR Master Mix (Kapa Biosystems, Merck KGaA, Darmstadt, Germany) on a PicoReal Real-Time PCR System (Thermo Scientific, Waltham, MA, USA). All reactions followed the temperature program recommended by the master mix manufacturer and were performed in duplicate. PCR specificity was verified by analysis of melting curves, and reaction efficiency for each gene was determined using standard curves, yielding values between 97% and 103%.

#### 4.3.4. Assessment of Expression Stability in the Selecting Reference Genes

NormFinder (version 0.953) and RefFinder (https://www.ciidirsinaloa.com.mx/RefFinder-master/, accessed on 21 October 2024), were employed, alongside geNorm and BestKeeper, to comprehensively evaluate the expression stability of the candidate HKGs.

To determine the most suitable reference genes, we applied the NormFinder algorithm [30] using the original Microsoft Excel add-in. In contrast to pairwise comparison models, NormFinder implements a model-based approach for estimating expression variance. The algorithm calculates a stability value derived from the combined assessment of intra-group variation (within biological or technical replicates) and inter-group variation (systematic differences between experimental conditions). Relative expression quantities were exported from PicoReal Software 2.2 (Thermo Scientific) and directly used as input for the analysis. A lower stability score indicates higher consistency of expression for the candidate reference gene.

To complement the NormFinder analysis, gene expression stability was further evaluated using the geNorm macro (geNorm v3.5), which assesses the consistency of expression ratios between all candidate reference genes [29]. The algorithm calculates an M-value for each gene based on the average pairwise variation with all other genes, where lower M-values indicate higher stability. Through stepwise elimination of the least stable genes, geNorm generates a ranked list of candidates and estimates the optimal number of reference genes required for reliable normalization.

Gene stability was additionally evaluated using BestKeeper [28], which evaluates candidate reference genes based on the variation in their raw Ct values. The algorithm calculates the standard deviation (SD) and coefficient of variation (CV) for each gene, with SD > 1 indicating instability. In this analysis, we also included the target gene *Fabp4* to examine correlation patterns relative to the candidate reference genes. BestKeeper further performs a pairwise correlation analysis between each gene and the BestKeeper Index, with correlation coefficients (r) closer to 1 indicating greater stability.

RefFinder, an online integrative platform combined the outputs of NormFinder, geNorm, BestKeeper, and the comparative ΔCt method, providing a unified evaluation system for reference gene stability. For each algorithm, RefFinder assigns an algorithm-specific rank to every candidate gene and subsequently calculates a geometric mean of these ranks to produce a final comprehensive stability index [31].

The stability of the tested reference genes was additionally evaluated using the comparative ΔCt method proposed by Silver et al. [32]. In this approach, each candidate reference gene is compared pairwise against all other candidates by calculating ΔCt values. For every pairwise comparison, the standard deviation (SD) of the ΔCt values is calculated across all samples in the dataset. These SD values are then averaged, and the gene with the lowest mean SD is considered the most stable, as it displays the least variability relative to the other reference genes.

By integrating model-based, pairwise, variance-oriented, and ΔCt-dependent analytical principles, this approach minimizes the influence of any single technique and yields a more balanced evaluation. As a result, the multi-algorithm framework provided a robust, consistent, and highly reliable assessment of reference gene suitability under the specific experimental conditions of the study.

### 4.4. Statistical Analysis

Statistical processing and data visualization were conducted using GraphPad Prism 10.5.0 Software LLC (Boston, MA, USA). Descriptive statistics were applied to determine the mean and standard deviation (SD) of ΔCt values, while the standard error of the mean (SEM) was calculated for raw Ct data. Normality was assessed using the Kolmogorov–Smirnov test. Normally distributed data were analyzed by one-way ANOVA with Tukey’s post hoc test, whereas non-normal data were evaluated using the Kruskal–Wallis test followed by Dunn’s post hoc comparisons. Statistical significance was set at *p* < 0.05.

## 5. Conclusions

Our findings strengthen the growing consensus that traditional reference genes cannot be assumed to be universally stable, particularly in systems undergoing pronounced metabolic and morphological changes. The 3T3-L1 differentiation model exemplifies such a system, in which the stability of HKGs is highly context-dependent and influenced by both the stage of adipogenesis and the lipid-modulating treatments applied. The multi-step evaluation strategy employed in this study—including algorithm-based approaches (geNorm, NormFinder, RefFinder, BestKeeper), the ΔCt method, correlation index with specific target genes (*Fabp4* and *Srebf1*), and assessment of intergroup variation—provided a more rigorous and reliable framework for identifying suitable HKGs. *Ppia*, *36b4*, and *B2M* emerged as the most robust reference candidates, collectively forming a stable and functionally diverse normalization panel.

This approach highlights the importance of empirically validating reference gene selection to ensure the accurate interpretation of transcriptional changes in adipogenic models.

## Figures and Tables

**Table 1:** Descriptive statistical evaluation of candidate reference genes using the BestKeeper algorithm.

	*Actb*	*Hprt*	*36b4*	*Hmbs*	*Ppia*	*Gapdh*	*Ywhaz*	*B2M*	*18S*
n	45	45	45	45	45	45	45	45	45
geo Mean [CP]	19.58	28.61	23.30	14.52	22.87	18.94	21.46	21.73	14.49
ar Mean [CP]	19.62	28.61	23.33	14.61	22.90	18.96	21.48	21.76	14.59
min [CP]	18.10	27.24	21.62	12.29	21.43	16.69	19.87	19.81	12.19
max [CP]	21.81	30.15	26.29	18.09	25.25	21.63	23.18	24.53	20.03
std dev [±CP]	0.98	0.47	1.06	1.38	0.91	0.84	0.69	1.01	1.46
CV [% CP]	5.00	1.63	4.54	9.48	3.98	4.41	3.19	4.62	10.03

**Table 2:** BestKeeper pairwise correlation and regression analysis of candidate reference genes.

	*Actb* vs. BK	*Hprt* vs. BK	*36b4* vs. BK	*Hmbs* vs. BK	*Ppia* vs. BK	*Gapdh* vs. BK	*Ywhaz* vs. BK	*B2M* vs. BK	*18S* vs. BK	*Srebf1* vs. BK	*Fabp4* vs. BK
coeff. of corr. [r]	0.910	0.456	0.962	0.988	0.973	0.664	0.900	0.941	0.969	−0.538	−0.472
coeff. of det. [r^2^]	0.828	0.208	0.925	0.976	0.947	0.441	0.810	0.885	0.939	0.289	0.221
intercept [CP]	1.607	23.694	2.502	−14.184	4.579	6.757	8.196	1.969	−16.150	37.056	43.105
slope [CP]	0.891	0.244	1.031	1.424	0.906	0.604	0.657	0.979	1.521	−0.503	−0.945
SE [CP]	±0.467	±0.546	±0.338	±0.257	±0.247	±0.783	±0.367	±0.406	±0.446	±0.907	±2.044
*p*-value	0.001	0.002	0.001	0.001	0.001	0.001	0.001	0.001	0.001	0.001	0.001
Power [x-fold]	1.85	1.18	2.04	2.68	1.87	1.52	1.58	1.97	2.87	0.71	0.52

**Table 3:** Pairwise ΔCt stability evaluation among all candidate reference genes.

Gene Names	Mean ΔCt	SD	Mean SD *	Gene Names	Mean ΔCt	SD	Mean SD *
*Actb/Hprt*	9.00	1.09		*Ppia/Actb*	3.28	0.50	
*Actb/36b4*	3.72	0.54		*Ppia/Hprt*	5.72	0.99	
*Actb/Hmbs*	5.01	0.79		*Ppia/36b4*	0.45	0.31	
*Actb/Ppia*	3.28	0.50		*Ppia/Hmbs*	8.29	0.71	
*Actb/Gapdh*	1.07	0.87		*Ppia/Gapdh*	3.93	0.90	
*Actb/Ywhaz*	1.87	0.50		*Ppia/Ywhaz*	1.42	0.51	
*Actb/B2M*	2.15	0.59		*Ppia/B2M*	1.13	0.42	
*Actb/18S*	5.02	0.96	0.73 ^ab^	*Ppia/18S*	8.30	0.93	0.66 ^b^
*Hprt/Actb*	9.00	1.09		*Gapdh/Actb*	1.07	0.87	
*Hprt/36b4*	5.28	1.16		*Gapdh/Hprt*	9.65	0.89	
*Hprt/Hmbs*	14.01	1.51		*Gapdh/36b4*	4.37	1.00	
*Hprt/Ppia*	5.72	0.99		*Gapdh/Hmbs*	4.36	1.29	
*Hprt/Gapdh*	9.65	0.89		*Gapdh/Ppia*	3.93	0.90	
*Hprt/Ywhaz*	7.13	0.84		*Gapdh/Ywhaz*	2.52	1.01	
*Hprt/B2M*	6.85	1.09		*Gapdh/B2M*	2.80	1.03	
*Hprt/18S*	14.02	1.64	1.15 ^a^	*Gapdh/18S*	4.34	1.43	1.05 ^ab^
*36b4/Actb*	3.72	0.54		*Ywhaz/Actb*	1.87	0.50	
*36b4/Hprt*	5.28	1.16		*Ywhaz/Hprt*	7.13	0.84	
*36b4/Hmbs*	8.73	0.63		*Ywhaz/36b4*	1.85	0.71	
*36b4/Ppia*	0.45	0.31		*Ywhaz/Hmbs*	6.88	0.96	
*36b4/Gapdh*	4.37	1.00		*Ywhaz/Ppia*	1.42	0.51	
*36b4/Ywhaz*	1.85	0.71		*Ywhaz/Gapdh*	2.52	1.01	
*36b4/B2M*	1.57	0.40		*Ywhaz/B2M*	0.56	0.46	
*36b4/18S*	8.74	0.86	0.70 ^ab^	*Ywhaz/18S*	6.89	1.15	0.77 ^ab^
*Hmbs/Actb*	5.01	0.79		*B2M/Actb*	2.15	0.59	
*Hmbs/Hprt*	14.01	1.51		*B2M/Hprt*	6.85	1.09	
*Hmbs/36b4*	8.73	0.63		*B2M/36b4*	1.57	0.40	
*Hmbs/Ppia*	8.29	0.71		*B2M/Hmbs*	7.16	0.75	
*Hmbs/Gapdh*	4.36	1.29		*B2M/Ppia*	1.13	0.42	
*Hmbs/Ywhaz*	6.88	0.96		*B2M/Gapdh*	2.80	1.03	
*Hmbs/B2M*	7.16	0.75		*B2M/Ywhaz*	0.56	0.46	
*Hmbs/18S*	0.01	0.43	0.88 ^ab^	*B2M/18S*	7.17	0.95	0.71 ^ab^
*18S/Actb*	5.02	0.96		*18S/Ppia*	8.30	0.93	
*18S/Hprt*	14.02	1.64		*18S/Gapdh*	4.37	1.43	
*18S/36b4*	8.74	0.86		*18S/Ywhaz*	6.89	1.15	
*18S/Hmbs*	0.22	0.36		*18S/B2M*	−7.17	0.95	1.04 ^ab^

* Differences between mean SDs were analyzed using one-way ANOVA with Tukey’s multiple-comparison test. Different superscript letters denote significant differences between groups (*p* < 0.05).

**Table 4:** Correlation coefficients (r) and *p*-values between candidate genes and *Fabp4*.

*Fabp4* vs.	*Actb*	*Hprt*	*36b4*	*Hmbs*	*Ppia*	*Gapdh*	*Ywhaz*	*B2M*	*18S*
n	*45*	*45*	*45*	*45*	*45*	*45*	*45*	*45*	*45*
Spearman (r) correlation	−0.56	0.27	−0.61	−0.41	−0.44	0.11	−0.32	−0.51	−0.39
*p*-value	<0.001	0.074	<0.001	0.006	0.003	0.492	0.033	<0.001	0.008

**Table 5:** Correlation coefficients (r) and *p*-values between candidate genes and *Srebf1*.

*Srebf1* vs.	*Actb*	*Hprt*	*36b4*	*Hmbs*	*Ppia*	*Gapdh*	*Ywhaz*	*B2M*	*18S*
n	*45*	*45*	*45*	*45*	*45*	*45*	*45*	*45*	*45*
Spearman (r) correlation	−0.65	−0.08	−0.62	−0.57	−0.54	−0.01	−0.51	−0.56	−0.54
*p*-value	<0.001	0.594	<0.001	<0.001	<0.001	0.966	<0.001	<0.001	<0.001

**Table 6:** Cross-method stability ranking of candidate reference genes.

	Mean ± SD	ΔCt (SD)	NormFinder	geNorm	BestKeeper (CV)	BestKeeper (r) Coefficient	RefFinder	Spearman (r) Coefficient
n	45	45	45	45	45	45	45	45
*Ppia*	22.89 ± 1.07	1	1	1	3	2	1	Weak to Moderate
*Ywhaz*	21.48 ± 0.84	5	5	4	2	7	4	Weak
*36b4*	23.33 ± 1.22	2	2	2	5	4	2	Moderate
*B2M*	21.76 ± 1.18	3	3	2	6	5	3	Weak
*Actb*	19.62 ± 1.11	4	4	3	7	6	5	Moderate
*Hprt*	28.61 ± 0.61	9	8	7	1	9	6	Non
*Hmbs*	14.61 ± 1.63	6	6	5	8	1	8	Weak to Moderate
*18S*	18.97 ± 1.04	7	7	6	9	3	9	Weak
*Gapdh*	18.97 ± 1.04	8	9	8	4	8	7	Non

**Mean Ct ± SD**—mean Ct value and standard deviation for each HKG across all samples. **ΔCt Method Rank**—stability ranking using the comparative ΔCt method. **NormFinder Rank**—based on combined intra- and inter-group variation (stability value). **geNorm Rank**—based on M value (average pairwise variation). **BestKeeper (statistic) Rank**—based on the average rank of standard deviation (SD) and coefficient of variation (CV%). **BestKeeper (regression) Rank**—based on the average rank of Pearson correlation coefficient (r), coefficient of determination (R^2^), and slope of regression from linear regression against the BestKeeper Index. **Spearman correlation (r) coefficient** between the mean Ct of each candidate reference gene and *Fabp4* or *Srebf1* expression. Abbreviation: Non (r < 0.30), Weak (0.30 ≤ r < 0.50), Moderate (0.50 ≤ r < 0.70). **ReFinder (consensus) Rank**—summary ranking based on geometric mean of individual gene ranks from ΔCt, geNorm, NormFinder, and BestKeeper Kruskal–Wallis.

**Table 7:** Design of experimental groups and timeline.

Stages/Groups	NC *	IC *		PA9/DHA9	PA18/DHA18
Day 0 (24 h)	Growth arrest at 100% confluence				
Day 1–2 (48 h)	Preadipocytes cultured in CM (NC0, NC9, NC18)	Induction in IM		Induction in IM + 60 µM PA/DHA	Induction in IM + 60 µM PA/DHA
Day 3–9		Preadipocyte maturation in MM (IC9)	Preadipocyte maturation in MM + 60 µM PA or DHA		Preadipocyte maturation in MM + 60 µM PA or DHA
Day 9–18		Mature adipocytes cultured in MM (IC18)		-	Mature adipocytes cultured in MM

* The digit after the abbreviation corresponds to the day of sample collection.

**Table 8:** Sequences of selected housekeeping and target genes used in RT-qPCR.

Abbreviation	Full Name	Forward Primer	Reverse Primer	Product Size (bp)
*B2M* NM_009735.3	Beta-2 microglobulin	TGTATGCTATCCAGAAAACCCCT	TTTCAATGTGAGGCGGGTGG	117
*Ppia* NM_008907.2	Peptidylprolyl isomerase A	GAACATTGTGGAAGCCATGGAG	AGATGGGGTAGGGACGCTC	163
*Gapdh* NM_001289726.2	Glyceraldehyde-3-phosphate dehydrogenase	CCCACTCTTCCACCTTCGAT	CTTGCTCAGTGTCCTTGCTG	181
*Ywhaz* NM_001356569.1	Tyrosine 3-monooxygenase/tryptophan 5-monooxygenase activation protein, zeta polypeptide	AGACGGAAGGTGCTGAGAAA	TTGTCATCACCAGCAGCAAC	211
*Hmbs* NM_001110251.1	Hydroxymethylbilane synthase	CCTGAAGGATGTGCCTACCA	CCACTCGAATCACCCTCATCT	175
*36b4* NM_007475.5	Ribosomal protein, large, P0	TTATAACCCTGAAGTGCTCGAC	CGCTTGTACCCATTGATGATG	147
*Hprt* NM_013556.2	Hypoxanthine guanine phosphoribosyl transferase	ACAGGCCAGACTTTGTTGGA	ACTTGCGCTCATCTTAGGCT	150
*Actb* NM_007393.5	β-actin	CCTCTATGCCAACACAGTGC	GTACTCCTGCTTGCTGATCC	211
*18S* NR_046271.1	18S ribosomal RNA	ATGCGGCGGCGTTATTCC	GCTATCAATCTGTCAATCCTGTCC	204
*Srebf1* NM_011480.4	Mus musculus sterol regulatory element binding transcription factor 1	TTGACACGTTTCTTCCTGAGC	CAGTTCAACGCTCGCTCTAG	239
*Fabp4* NM_024406.4	Fatty acid-binding protein 4	AACTGGGCGTGGAATTCGAT	CCACCAGCTTGTCACCATCT	150

## Data Availability

The original contributions presented in this study are included in the article. Further inquiries can be directed to the corresponding author.

## Abbreviations

The following abbreviations are used in this manuscript:

*18S*	18S ribosomal RNA
*36b4*	Ribosomal protein, large, P0
3T3-L1	Mouse embryonic fibroblast cell line used as an adipogenesis model
*Actb*	β-actin
*B2M*	Beta-2 microglobulin
BK	BestKeeper index
cDNA	Complementary deoxyribonucleic acid
CM	Control medium
Ct	Cycle threshold
ΔCt	delta Ct
CV	coefficient of variation
DHA	Docosahexaenoic acid
DMEM	Dulbecco’s Modified Eagle’s Medium
*Fabp4*	Fatty acid-binding protein 4
*Gapdh*	Glyceraldehyde-3-phosphate dehydrogenase
HKG	Housekeeping genes
*Hmbs*	Hydroxymethylbilane synthase
*Hprt*	Hypoxanthine guanine phosphoribosyl transferase
IBMX	3-Isobutyl-1-methylxanthine
IC	Differentiated adipocytes (positive control)
MM	Adipocyte maintenance medium
MHC I	Major histocompatibility complex class I
NC	Undifferentiated control preadipocytes (negative control)
PA	Palmitic acid
PBS	Phosphate-buffered saline
*Ppia*	Peptidylprolyl isomerase A
RNA	Ribonucleic acid
RT-qPCR	Reverse transcription quantitative polymerase chain reaction
SD	standard deviation
SEM	Standard error of the mean
*Srebf1*	Sterol regulatory element binding transcription factor 1
*Ywhaz*	Tyrosine 3-monooxygenase/tryptophan 5-monooxygenase activation protein, zeta polypeptide
ORO	Oil Red O stain
